# Methylenetetrahydrofolate Reductase (MTHFR) C677T Mutation and Complete Hemolysis, Elevated Liver Enzymes, and Low Platelets (HELLP) Syndrome With Rare Complications: A Case Report

**DOI:** 10.7759/cureus.114797

**Published:** 2026-08-19

**Authors:** Gaurav Chitkara, Nazir Ahmad Pala, Rakesh K Koul, Hilal Bhat, Maqsood Ahmad Dar

**Affiliations:** 1 Department of Internal Medicine, Maharaja Agrasen Medical College, Agroha, IND; 2 Department of Internal Medicine, Government Medical College, Srinagar, IND

**Keywords:** exudative retinal detachment, hellp, mthfr c677t, preeclampsia, stroke

## Abstract

A severe obstetric condition presenting with hemolysis and elevated liver enzymes, along with a low platelet count, is defined by the abbreviation HELLP, presenting hazardous complications to both mother and fetus. HELLP can be diagnosed in the antepartum period. For accurate diagnosis, biomarkers play an important role, as blood pressure is not directly linked to the severity of the disease, even though it is described as a hypertensive disorder. We detail the case of a 30-year-old primigravida from a tribal region in Jammu and Kashmir, India, presenting at 24 weeks of gestation. Although she did not have hypertension, she developed full HELLP syndrome, which included hemolysis, elevated liver enzymes, and low platelet count. Her clinical course was notably complicated by multiterritorial cerebral infarctions and bilateral exudative retinal detachment. Further tests showed normal folate and homocysteine levels but also confirmed the presence of a heterozygous methylenetetrahydrofolate reductase (MTHFR) C677T mutation. There was healing of the retinal detachment once pregnancy was terminated, thereby eliminating the need for ophthalmological surgery. Therefore, this case highlights the need to further study the contribution of the MTHFR polymorphism in persons suffering from HELLP syndrome.

## Introduction

First identified by Weinstein [1] in 1982, hemolysis, elevated liver enzymes, and low platelets (HELLP) syndrome is classified in the International Classification of Diseases, 11th Revision, as a multisystem form of preeclampsia associated with adverse outcomes. HELLP occurs in about 0.5%-0.9% of pregnancies [2]. In 70% of cases, diagnosis occurs antepartum, before 27 weeks; in 10% of cases, 70% occur between 27 and 37 weeks of gestation; in 20% of cases, it occurs close to term; and in 30% of cases, it is diagnosed after delivery [2,3].

For accurate diagnosis, biomarkers play an important role, as blood pressure (BP) is not directly linked to disease severity, even though it is described as a hypertensive disorder. The studies by Sibai [4] and Martin et al. [5] concluded that there was minimal or total absence of hypertension and proteinuria. Specifically, according to findings in confirmed cases by Sibai, about 10%-15% are absent of these signs, whereas Martin et al. confirmed, based on their study, that without systolic and diastolic pressure elevation (≥90 mmHg), signs of patient suffering from HELLP syndrome with thrombocytopenia (<100 × 10^9^/L) were present [4,5].

Onset is mostly abrupt and difficult to predict, as there are a number of maternal risk factors involved, such as advanced age (>30 years), obesity, preexisting diabetes, chronic hypertension, and previous HELLP syndrome cases or anomalies of fetal placentation [6,7]. Furthermore, maternal risk is enhanced by biochemical markers such as low high-density lipoprotein cholesterol, hyperuricemia, and hypertriglyceridemia [8].

During pregnancy, there is a crucial interaction among immune, endocrine, and metabolic systems, resulting in healthy placentation, nourishment, and immune balance. Various pregnancy-related disorders, such as HELLP syndrome, were observed when disruptions in these interactions occurred, affecting hemolysis, elevated liver enzymes, and low platelet counts [9]. Among multiple factors, genetic and environmental factors play a crucial role in the development of the syndrome [10].

This life-threatening condition presents significant diagnostic challenges and leads to severe complications in 12.5%-65% of patients, with maternal mortality rates reaching as high as 30%. Documented complications include acute kidney injury (AKI), liver damage, disseminated intravascular coagulation, sepsis, acute respiratory distress syndrome, preterm delivery, and stroke [2,3,7]. Physiological changes during pregnancy increase cerebrovascular risk, with an incidence of 11-34 strokes per 100,000 deliveries. Most strokes occur around the time of childbirth or after (90%), while only 10% happen before labor. Causes of pregnancy-related strokes include HELLP syndrome, posterior reversible encephalopathy syndrome, eclampsia, thrombotic microangiopathies (TMAs; thrombotic thrombocytopenic purpura (TTP) or hemolytic uremic syndrome (HUS)), and thrombophilias [11]. Cerebral thrombosis associated with HELLP syndrome is extremely rare. Managing it presents a clinical challenge: anticoagulation is required to treat the thrombosis, but this is complicated by the patient's increased bleeding risk caused by severe thrombocytopenia [12].

## Case presentation

A 30-year-old female patient from a tribal community in Jammu and Kashmir, India, who is a primigravida and a product of consanguineous marriage, presented at 24 weeks of gestation with complaints of sudden onset of blurred vision in both eyes persisting for 24 hours at a peripheral hospital. Ophthalmology consultation indicated visual acuity of counting fingers at 0.5 meters in both eyes. Fundus examination identified bilateral exudative macular retinal detachment.

The patient was referred to the obstetrics department of Srinagar Government Medical College Hospital for early termination of pregnancy. Obstetrics ultrasonography at this point revealed a single, live, intrauterine fetus of gestational age 22-23 weeks with breech presentation. At 24 weeks gestation, medical termination of pregnancy (MTP) was carried out by medical induction, resulting in vaginal delivery of a nonviable fetus two days after the onset of visual complaints. Twenty-four hours after MTP, the patient developed right-sided body weakness and motor aphasia. In view of such complaints, the patient was admitted to the medicine department of Srinagar Government Medical College Hospital for evaluation and management.

On admission, the patient was conscious, cooperative, and responding to verbal commands, with a BP of 116/78 mmHg, PR of 97 bpm, peripheral oxygen saturation of 98% on room air, and respiratory rate of 18 breaths/min. On examination, significant pallor was noted, and icterus was noted in the sclera. However, clubbing, cyanosis, pedal edema, and lymphadenopathy were absent, and jugular venous pressure was not raised. Respiratory, cardiovascular system, and abdominal examinations were unremarkable. Central nervous system examination revealed a power of 1/5 in the right upper and lower limbs, both proximal and distal, and 5/5 on the left side.

Bulk and tone appeared to be normal. There was subtle right facial paralysis. She was unable to vocalize but able to comprehend, suggesting motor aphasia. Sensations were intact; however, cortical sensations could not be assessed due to aphasia. Deep tendon reflexes were 2+ all over; the right plantar was mute, and the left plantar showed a flexor response.

**Figure 1:**
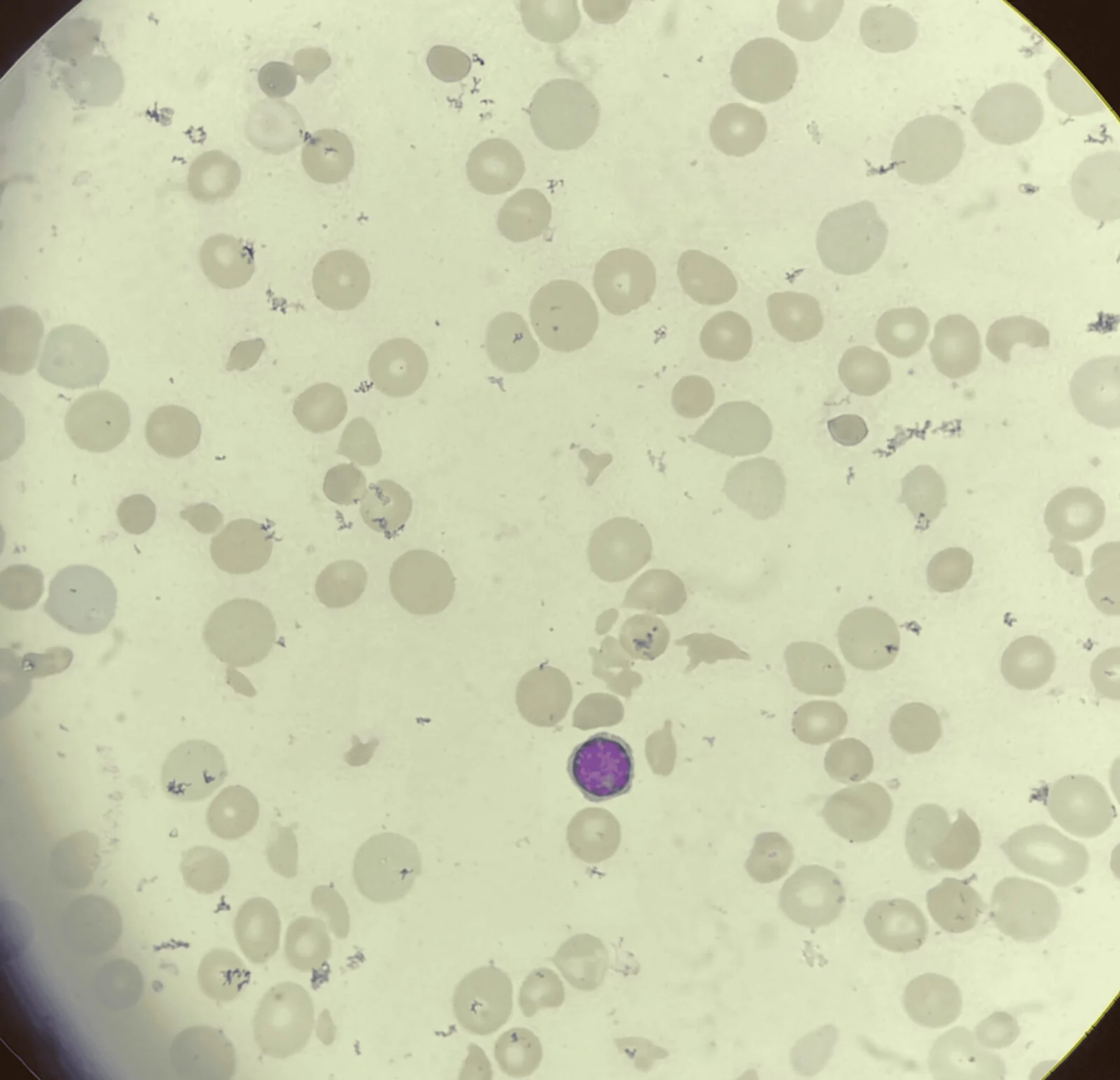
Peripheral blood film of the patient showing reduced RBC density, marked anisopoikilocytosis with a normocytic to macrocytic picture. Polychromasia, nucleated red blood cells, a few spherocytes, and schistocytes are also noted. Features suggestive of hemolysis RBC: red blood cell

The patient's antepartum lab parameters before MTP were as follows: Hb of 6.3 g/dL, total leukocyte count of 10,400/µL, platelet count of 13,000/µL, peripheral blood film suggestive of microangiopathic hemolytic anemia (Figure 1), lactate dehydrogenase >4,500 U/L, aspartate aminotransferase of 135 U/L, alanine transaminase of 62 U/L, and total serum bilirubin of 2.4 mg/dL, favoring a diagnosis of complete HELLP syndrome (Mississippi Class I) [5]. The kidney function test results are as follows: urea level of 103 mg/dL and serum creatinine of 1.7 mg/dL. The coagulogram prothrombin time, international normalized ratio, and activated partial thromboplastin time pre- and post-MTP were normal. D-dimer was elevated at 6.1 mg/dL (<0.3 mg/dL). The direct Coombs test and indirect Coombs tests were negative. Erythrocyte sedimentation rate was 64 mm/hour, and C-reactive protein was positive (36 mg/L). Iron profile, vitamin B12, and folate levels were normal. Urine examination revealed 2+ protein with a urine albumin-to-creatinine ratio of 558.63 mg/g. She had hypocalcemia with corrected calcium levels of 7.74 mg/dL due to low vitamin D levels (7.8 ng/mL). Her uric acid was raised (10.9 mg/dL), and triglycerides were elevated (470 mg/dL) (Table 1).

**Table 1:** Antepartum and postpartum blood investigations of the patient HCT: hematocrit; MCV: mean corpuscular volume; MCH: mean corpuscular hemoglobin; TLC: total leukocyte count; PLT: platelet; AST: aspartate aminotransferase; ALT: alanine aminotransferase; ALP: alkaline phosphatase; LDH: lactate dehydrogenase

Parameter	Reference ranges of parameter	Antepartum	Day 1 postpartum	Day 3 postpartum	Day 10 postpartum
Hb (g/dL)	11.0-14.0	6.3	4.8	6.2	8.9
HCT (%)	31-41	19.6	15.2	20.1	25.1
MCV (fL)	80-100	95.1	102.0	108.1	110.1
MCH (pg)	27-33	30.6	32.2	33.3	32.9
TLC (per µL)	4,000-11,000	10,400	20,600	7,950	6,760
PLT (per µL)	150-450 x 10^3^	13,000	27,000	21,000	74,000
Urea (mg/dL)	15-45	103	68	51	31
Creatinine (mg/dL)	0.4-0.8	1.7	1.6	1.25	0.91
Na^+^ (mEq/L)	130-145	134	140	140	137
K^+^ (mEq/L)	3.3-5.0	4.6	3.2	3.5	4.2
Uric acid (mg/dL)	2.0-6.3	10.9	-	-	2.3
Corrected Ca^2+^ (mg/dL)	8.2-9.7	7.74	-	-	9.12
Total bilirubin (mg/dL)	0.1-1.1	2.4	2.7	2.8	1.0
AST (U/L)	3-35	135	237	247	62
ALT (U/L)	7-41	62	83	159	27
ALP (U/L)	30-147	173	172	115	75
Total protein (g/dL)	5.6-7.6	7.1	6.9	6.1	6.0
Albumin (g/dL)	3.5-5.5	3.3	3.0	2.7	3.1
LDH (U/L)	115-211	>4,500	-	-	1,945
Triglycerides (mg/dL)	<150	470	-	-	210

**Figure 2:**
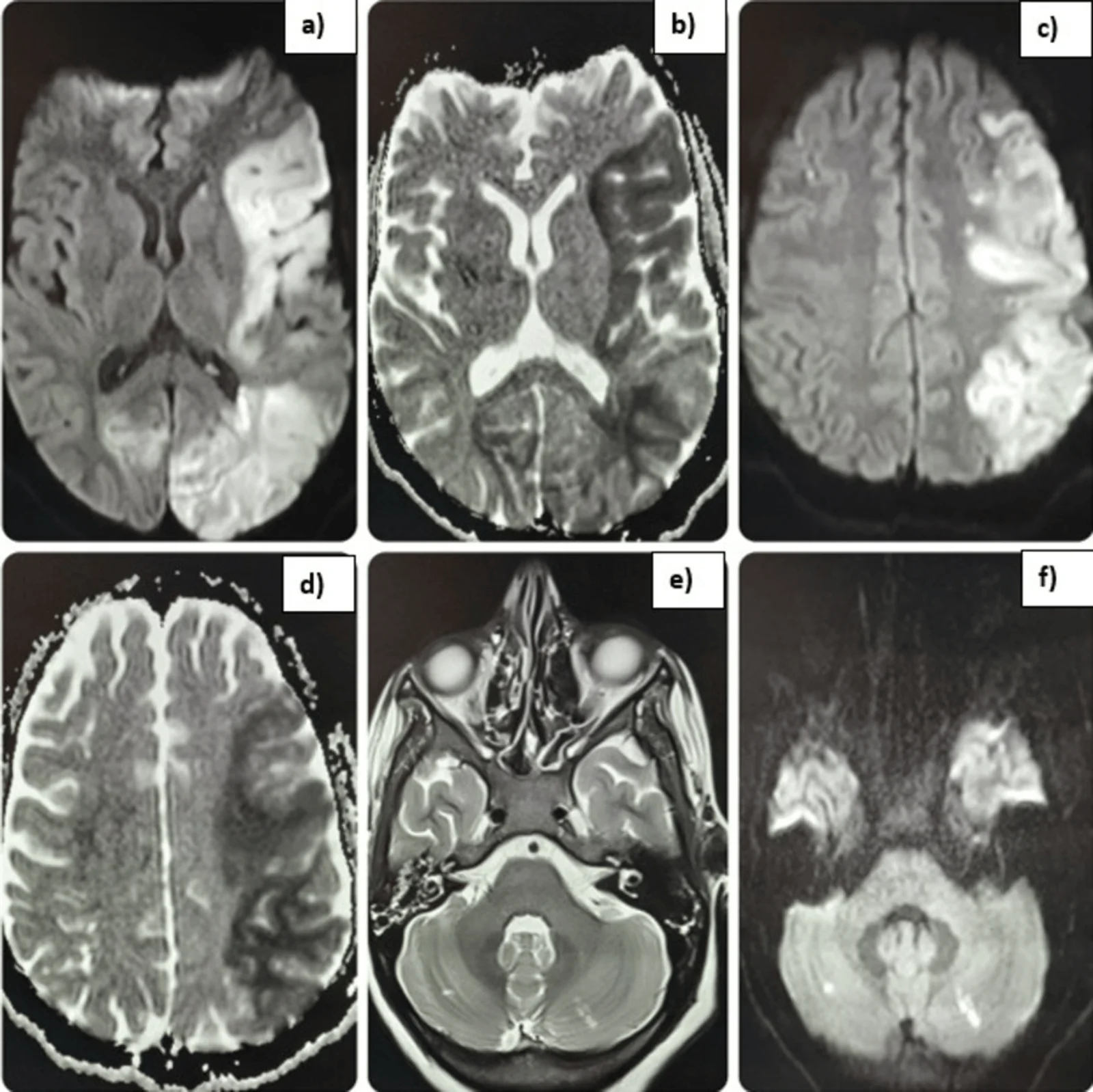
MRI brain demonstrating multifocal acute ischemic infarctions with diffusion restriction. (a) Axial DWI demonstrates a large area of diffusion restriction involving the left parieto-temporo-occipital region and smaller similar areas in the right occipital region, suggestive of acute infarction in the left middle cerebral artery and bilateral posterior cerebral artery territories. (b) Corresponding ADC map shows low signal intensity in the same region, confirming restricted diffusion consistent with acute infarction. (c) Axial DWI at a higher level demonstrates additional areas of diffusion restriction involving the left parietal and occipital lobes. (d) Corresponding ADC map confirms restricted diffusion in the involved cerebral regions. (e) Axial T2-weighted image demonstrates hyperintense signal changes involving bilateral cerebellar hemispheres. (f) Axial DWI demonstrates bilateral cerebellar diffusion restriction, consistent with acute infarcts in bilateral posterior inferior cerebellar artery territories MRI: magnetic resonance imaging; DWI: diffusion-weighted imaging; ADC: apparent diffusion coefficient

**Figure 3:**
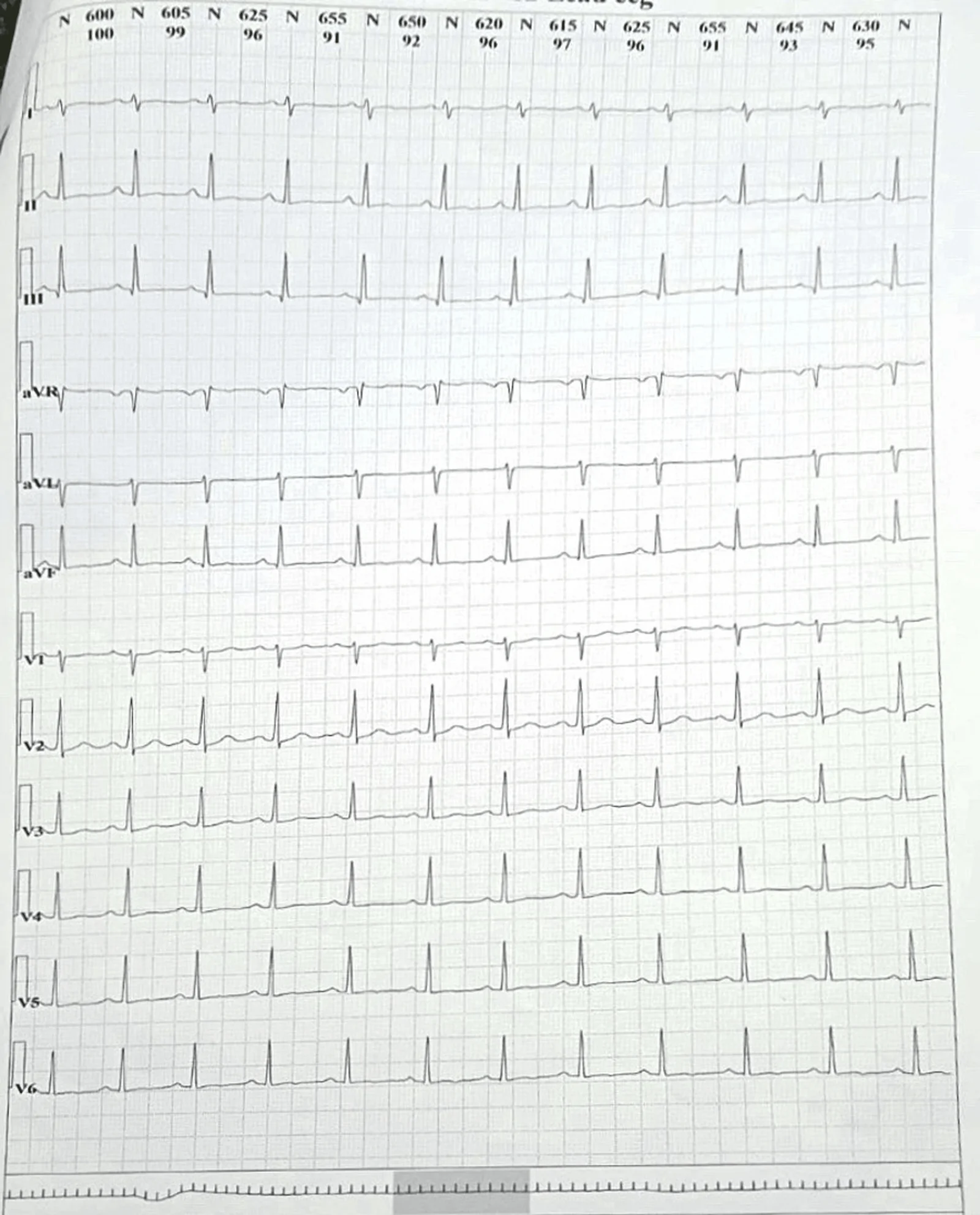
24-hour Holter image for RD and stroke workup aVR: augmented vector right; aVL: augmented vector left; aVF: augmented vector foot; RD: retinal detachment

The noncontrast computed tomography revealed a hypodensity in the left frontoparietal region, likely representing an acute infarct. Magnetic resonance imaging of the brain showed a large diffusion restriction area involving the left parieto-temporo-occipital lobe and smaller similar areas in the right occipital lobe and bilateral cerebellar hemispheres, representing acute infarcts, suggestive of left middle cerebral artery, bilateral posterior cerebral artery, and B/L posterior inferior cerebellar artery territory acute infarcts (Figure 2). For retinal detachment and stroke workup, 2D echo and 24-hour Holter monitoring were normal (Figure 3). The vasculitic profile: antinuclear antibody and antineutrophil cytoplasmic antibody (perinuclear antineutrophil cytoplasmic antibody and cytoplasmic antineutrophil cytoplasmic antibody) were negative. The antiphospholipid antibody profile (lupus anticoagulant, anticardiolipin Ab IgM and IgG) was negative. Hepatitis B, hepatitis C, and retroviral serology were negative; quantitative rapid plasma reagin was nonreactive (Table 2). Thrombophilic profile-antithrombin III and protein C levels were normal; free protein S was slightly reduced. Amplification-refractory mutation system polymerase chain reaction test was carried out for the analysis of genetic factors such as Factor V Leiden mutation and Prothrombin G20210A mutation. The absence of these mutations indicated that both genes were normal and did not carry a mutation. However, the presence of a heterozygous methylenetetrahydrofolate reductase (MTHFR) C677T mutation indicated that one gene was normal, whereas the other carried a mutation; however, per American College of Medical Genetics and Genomics criteria, this gene is commonly present as a low-penetration variant of a functional polymorphism in the general population. Hence, it cannot be used to diagnose inherited thrombophilia (Table 3).

**Table 2:** Serological findings of the patient and their interpretations ANA: antinuclear antibody; p-ANCA: perinuclear antineutrophil cytoplasmic antibody; c-ANCA: cytoplasmic antineutrophil cytoplasmic antibody; APLA: antiphospholipid antibodies; RPR: rapid plasma reagin

Serological test	Findings	Interpretation
Vasculitic profile (ANA, p-ANCA, c-ANCA)	Negative	There was no evidence to support autoimmune vasculitis as a cause of retinal and cerebral complications
APLA profile (lupus anticoagulant, anticardiolipin Ab IgM and IgG)	Negative	No evidence was present supporting antiphospholipid syndrome as an etiology of stroke
Hepatitis B, Hepatitis C, and retroviral serology	Negative	Infectious vasculitic etiologies were excluded, leading to retinal detachment and stroke
Quantitative RPR	Nonreactive	Syphilitic vasculopathy was unlikely

**Table 3:** Genetic mutation findings of the patient and their interpretations ARMS-PCR: amplification-refractory mutation system polymerase chain reaction

Genetic marker	Results	Method of testing	Interpretations
MTHFR C677T	Heterozygous mutation present	ARMS-PCR	60%-70% of normal activity is retained; plasma homocysteine levels are normal, especially in individuals with adequate folate status, and this variant is considered to have minimal clinical effect
Factor V Leiden	Homozygous wild	ARMS-PCR	Both alleles are normal; no Factor V Leiden mutation detected
Prothrombin G20210A	Homozygous wild	ARMS-PCR	Both alleles are normal; no prothrombin G20210A mutation was detected

On the fifth postpartum day, fundus examination showed spontaneous reattachment of the retina in both eyes, without any ophthalmologic intervention, with improvement in her vision. During her hospital stay, three units of packed red cells transfusion and other supportive treatment were given along with physiotherapy. Regular BP monitoring was done, and BP was found to be normal on all occasions. Upon improvement of her lab parameters by the 10th postpartum day, she was discharged in a hemodynamically stable condition with contraceptive counseling and risks associated with subsequent pregnancy being explained. There was an apparent history of the demise of her sister with similar complaints in her seventh month of pregnancy. The clinical timeline of the events is shown in Table 4.

**Table 4:** Timeline summarizing the clinical course of the patient's condition RD: retinal detachment; GMC: Government Medical College; USG: ultrasonography; MTP: medical termination of pregnancy; HELLP: hemolysis, elevated liver enzymes, and low platelets; NCCT: noncontrast computed tomography; MRI: magnetic resonance imaging; ARMS-PCR: amplification-refractory mutation system polymerase chain reaction; MTHFR: methylenetetrahydrofolate reductase; PLT: platelet; AST: aspartate aminotransferase; ALT: alanine aminotransferase; Hb: hemoglobin

Timepoint	Event
24 weeks gestation (day 0)	Sudden bilateral blurred vision; CF 0.5m both eyes; bilateral exudative macular RD on fundus exam
Day 0	Referred to Obstetrics, GMC Srinagar; USG: single live fetus, 22-23 weeks, breech
Day 2 (24 weeks gestation)	MTP by medical induction; vaginal delivery of nonviable fetus
Day 3 (24 hour post-MTP)	Right-sided weakness + motor aphasia; admitted to Medicine; labs → Complete HELLP (Mississippi Class I); NCCT/MRI → multiterritorial infarcts; ARMS-PCR → heterozygous MTHFR C677T
Day 1 postpartum	Hb 4.8, PLT 27,000, AST/ALT rising
Day 3 postpartum	Platelet (21,000); AST/ALT peak
Day 5 postpartum	Spontaneous bilateral retinal reattachment
Day 10 postpartum	PLT 74,000, Hb 8.9; discharged stable

## Discussion

HELLP syndrome operates as a progressive, pregnancy-specific hepatic disorder carrying substantial risks of maternal mortality and severe morbidity. Diagnosis may hinge exclusively on biochemical alterations or a blend of laboratory and clinical findings [7]. Although affecting only a small fraction of all pregnancies (0.2%-0.9%), it appears as a secondary complication in up to 80% of preeclampsia cases. An analysis performed by Sibai among 1,117 patients depicted that only 12%-18% of people suffer from a normotensive condition, and the condition is referred to as “atypical HELLP” [4]. Patients suffering from HELLP syndrome commonly present with nonspecific symptoms such as malaise, headache, nausea, vomiting, and epigastric discomfort. Right upper abdominal tenderness is a more common clinical finding, reported in approximately 90% of cases. Edema, in contrast, has less diagnostic utility because it affects nearly one-third of normal pregnancies [7]. In the following case study, despite fulfilling the criteria regarding Mississippi Class I HELLP and having proteinuria, the patient was completely normotensive and did not present with the usual symptoms of HELLP syndrome.

With an exaggerated immune response in pregnant women, there is endothelial cell dysfunction. When typical preeclampsia was compared with HELLP syndrome, abnormal placentation was observed, resulting in increased blood flow among pregnant women, especially during 15 and 20 weeks after placentation of the fetus [6]. Von Willebrand factor was released in case of damage to the endothelium, leading to thrombocytopenia. Hulstein et al. and Shen et al., in their research, stated that there will be an initiation of an endothelial cascade from abnormal placental trophoblast debris circulating in blood [13,14]. The presence of microthrombi affected by signaling pathways as well as noncoding RNAs was seen in HELLP syndrome, similar to TMAs [10].

Studies suggested that both fetal and maternal genes are responsible for the occurrence of the syndrome, not just one gene as stated by the National Institutes of Health notes, but these results were different from the results of studies carried out on inherited thrombophilias [7]. A cohort study, performed in 2008, stated that mutations in Factor V Leiden, along with the prothrombin gene, were responsible for an increased risk of HELLP syndrome, irrespective of the syndrome being linked to heterozygosity for MTHFR [15]. The studies carried out by Nagy et al. and Demir et al. showed contrasting results, stating that HELLP syndrome is caused by a relation between MTHFR polymorphism and pregnant women having hypertension [16,17]. It has been noticed that MTHFR polymorphisms lead to hyperhomocysteinemia, but the pregnant women in the following study, irrespective of having multiterritorial infarcts, depicted normal levels of homocysteine. This finding in the present study indicated that, though the heterozygous MTHFR C677T mutation had been identified in our patient, it does not appear to be a positive contributor to cerebrovascular and ocular complications.

Many studies have been carried out in the past indicating a relationship between MTHFR polymorphism and adverse outcomes of pregnancy, but the latest studies and guidelines point to the fact that heterozygous C677T is a common variant and cannot contribute to inherited thrombophilia [18]. Hence, it can be stated that patients suffering from stroke as well as bilateral retinal detachment are a result of endothelial injury, TMA, vasospasm, and coagulation deformities associated with HELLP syndrome instead of MTHFR variants. Many current guidelines from professional organizations do not consider MTHFR to be a diagnostic marker for predicting HELLP syndrome, such as the American Society of Hematology [19] and the British Society of Hematology's [20] evaluation of inherited thrombophilias; they do not coindicate patients for evaluation. Similarly, the American College of Obstetricians and Gynecologists [21] organization, for the testing of thrombophilia or adverse pregnancy outcomes, does not involve testing of the MTHFR variant.

Proper clinical diagnosis is very essential, as any delay regarding diagnosis can severely affect the pregnancy outcomes. The diagnosis is also very essential, as some conditions such as acute fatty liver of pregnancy (AFLP), lupus flares, and renal TMAs have similar findings as seen in HELLP syndrome. AFLP is distinctly identified by hypoglycemia, a feature not present in HELLP. Although atypical HUS (often postpartum) and TTP (usually in the second or third trimester) show severe thrombocytopenia and microangiopathy, their liver involvement is typically milder than the significant transaminitis observed in HELLP. Similarly, antiphospholipid syndrome can resemble liver dysfunction but is primarily characterized by recurrent fetal loss and unprovoked thrombosis. Using this framework, our team was able to confidently exclude these other diagnoses [2,22].

AKI occurs in up to 15% of HELLP cases, whereas it is seen in only 1% of isolated preeclampsia [22]. Ocular complications are quite rare; retinal detachment affects only 0.9% of HELLP patients and up to 2% of those with severe preeclampsia [23]. Exudative retinal detachment occurs when failure of the tight junctions in the retinal pigment epithelium disrupts the outer blood-retinal barrier, leading to an accumulation of subretinal fluid. Typically presenting in third-trimester primiparous patients, it is an urgent indication for immediate fetal delivery to salvage vision. There were only six pieces of literature evidence suggesting that HELLP syndrome is responsible for bilateral exudative retinal detachment. It has been observed that, after delivery, reattachment and visual recovery occur within weeks in affected individuals, thereby providing reassurance [24]. There was hardly any evidence reporting normotensive complete HELLP syndrome and a heterozygous MTHFR C677T mutation showing clinical findings related to bilateral exudative retinal detachment and multiterritorial cerebral infarction. Patients should be educated regarding the good prognosis as well as the natural course of disease, which helps guide the patient and eliminate unnecessary worry [23].

HELLP syndrome even after delivery depicted increased levels of liver enzymes along with a decrease in platelet levels. During the postpartum period, within 23 hours after delivery, there is a decrease in platelet count to its lowest level, and within two days, a rise in HELLP symptoms is observed [7].

Within 23-48 hours after delivery, there can be a decline in platelet count and elevation of transaminases; hence, close monitoring is necessary for HELLP syndrome patients. An increase in platelet count was observed on the third day, and by the sixth day, it reaches up to 100,000/mm³ by the natural recovery course. There can be an increased risk of multiorgan failure if 96 hours of rebound were not achieved. Chances of severe complications occur if uric acid levels surpass 7.8 mg/dL [3]. Delayed recovery was observed in the patient in the following study, indicating that by the 10th day, the platelet count stabilizes. Key treatments included immediate removal of the placenta and fetus, magnesium sulfate to prevent seizures, BP management, and transfusions as needed. For future family planning, the recurrence risk of hypertensive disorders is 20.7%, increasing to 36.3% considering their history, but the absolute risk for strict HELLP syndrome stays relatively low at 7.2% [25].

This case report discusses a woman pregnant for the first time, with significant risk factors and a family history, who developed an atypical and severe HELLP syndrome, resulting in the development of rare complications such as bilateral exudative macular retinal detachment and a stroke. This led to the only option left: to terminate the pregnancy, thereby relieving retinal detachment on the fifth day, and by the 10th day postpartum, a rise in platelet count was observed. HELLP syndrome can be managed in various ways, such as controlling seizures, administering intravenous magnesium sulfate, prescribing antihypertensives for BP control, promptly delivering the fetus and placenta, and, if needed, including blood transfusions in the treatment plan [2,7].

Further testing of women showed that her syndrome can be worsened as she was carrying a heterozygous MTHFR C677T mutation gene, which is considered to be a valuable suspect for causing preeclampsia and HELLP syndrome, but, at present, evidence supporting this is inconsistent. At present, all reports suggest that the homozygous (TT) gene had a stronger association than the heterozygous (CT) gene, and similarly, current guidelines do not support MTHFR testing for HELLP syndrome. Therefore, the heterozygous C677T gene detected in our patient must be interpreted with caution and should not be concluded as the primary cause of disease.

## Conclusions

This interesting case report emphasizes that HELLP syndrome is an obstetric emergency that poses a great risk for mother and child and can lead to fatal complications. Less is known about its pathogenesis and the relationship between MTHFR polymorphism and HELLP syndrome. Genetic and environmental factors play an important role in the development of the syndrome, but the following study failed to correlate genetic factors, such as the MTHFR polymorphism connection, with the syndrome and also presented various ways by which the syndrome can be managed. The study highlighted the importance of the following research in the case of HELLP syndrome, such as the occurrence of cerebral infarctions, detachment of the bilateral retina, and the influence of MTHFR mutations.
